# Reducing primary cesarean delivery rate through implementation of a smart intrapartum surveillance system

**DOI:** 10.1038/s41746-023-00867-y

**Published:** 2023-07-11

**Authors:** Po Jen Cheng, You Hung Cheng, Steven S. W. Shaw, Hung Chi Jang

**Affiliations:** 1 Department of Obstetrics and Gynecology, Chang Gung Memorial Hospital-Linkou Medical Center, Chang Gung University College of Medicine, Taoyuan, Taiwan; 2 College of Medicine, Chang Gung University, Taoyuan, Taiwan; 3 Bone and Joint Research Center, Department of Orthopaedic Surgery, Chang Gung Memorial Hospital-Linkou, Taoyuan, Taiwan; 4 Graduate Institute of Biomedical Electronics and Bioinformatics College of Electrical Engineering and Computer Science National Taiwan University, Taipei, Taiwan; 5 Hongchi Women & Children’s Hospital, Taoyuan, Taiwan

**Keywords:** Quality of life, Health care

## Abstract

The rapid changes in clinical maternity situations that occur in a labor and delivery unit can lead to unpredictable maternal and newborn morbidities. Cesarean section (CS) rate is a key indicator of the accessibility and quality of a labor and delivery unit. This retrospective cross-sectional study assesses the nulliparous, term, singleton, vertex (NTSV) cesarean delivery rates before and after the implementation of a smart intrapartum surveillance system. Research data were collected from the electronic medical records of a labor and delivery unit. The primary outcome was the CS rate of the NTSV population. The data of 3648 women admitted for delivery were analyzed. Of the studied deliveries, 1760 and 1888 occurred during the preimplementation and postimplementation periods, respectively. The CS rate for the NTSV population was 31.0% and 23.3% during the preimplementation and postimplementation periods, respectively, indicating a significant 24.7% (*p* = 0.014) reduction in CS rate after the implementation of the smart intrapartum surveillance system (relative risk, 0.75; 95% confidence interval, 0.71–0.80). In the NTSV population, the vaginal and CS birth groups, no significant difference in terms of newborn weight, neonatal Apgar scores, composite neonatal adverse outcome indicator, and the occurrence of the following: neonatal intensive care unit admission, neonatal meconium aspiration, chorioamnionitis, shoulder dystocia, perineal laceration, placental abruption, postpartum hemorrhage, maternal blood transfusion, and hysterectomy before and after the implementation of the smart intrapartum surveillance system. This study reveals that the use of the smart intrapartum surveillance system can effectively reduce the primary CS rate for low-risk NTSV pregnancies without significantly affecting perinatal outcomes.

## Introduction

For women, childbirth is the most common reason for hospital admission. During hospitalization in a labor and delivery unit, rapid changes in clinical maternity situations can lead to unpredictable maternal and newborn mortalities and morbidities. A global analysis reported that almost half of all maternal deaths and one-third of all neonatal deaths occurred during the first 24 h of birth^1^. A delayed diagnosis and the late initiation of an effective treatment have contributed to numerous intrapartum maternal fetal deaths. Therefore, hospital labor and delivery units are advised to adopt the Maternal Early Warning Criteria (MEWC) to avoid delays in identifying and addressing obstetrical complications^2^. Various maternal early warning systems (MEWSs) based on the MEWC were developed and implemented in labor and delivery units. MEWSs facilitate early bedside evaluation, the timely recognition of intrapartum complications, and the implementation of prompt interventions for such complications. However, various obstacles can lead to MEWS malfunction^3^; they include logistic difficulties associated with paging multiple services, interdisciplinary collaboration, individual cognitive bias, and judgment errors. To overcome the aforementioned problems, reduce the workload associated with manual data entry, and reduce warning alarm fatigue, all of which are problems that often affect traditional MEWSs, we designed a smart intrapartum surveillance system that automatically reminds staff members to apply the MEWC and intrapartum care bundles for women in labor. In addition, we incorporated a fetal heart rate interpretation algorithm and electronic partogram (based on the revised definition of normal labor progress) into the smart intrapartum surveillance system. In the labor and delivery setting, the smart intrapartum surveillance system was merged with a Smart Birth Center (GeneJet., New Taipei City, Taiwan) architecture. This novel electronic maternal, fetal, and labor progress surveillance and early warning system was developed by iWard software (Advantech, Taipei, Taiwan); its algorithms can be used to assess maternal fetal vital signs and labor course, and it can automatically alert obstetricians regarding deviations from established safety ranges through a nursing control station, a nursing station dashboard, and Smart Birth Center patient bedside information terminals.

Cesarean section (CS) rate is a key indicator of the accessibility and quality of a labor and delivery unit^4,5^. Variations in hospital intrapartum practices can considerably influence CS rates. High CS rate has always been an important maternal health issue in Taiwan^6^. Since the 1990s, the CS rate in Taiwan has been reported as one of the leading countries in the world. The CS rate was approximately 33.1 to 37.9% from 2004 to 2021, which was significantly higher than the rate, 10–15%, that the World Health Organization (WHO) considers reasonable^7^. Almost all Taiwanese women give birth in hospitals, especially medium-sized maternity hospitals. Maternal preference played a minor part in explaining high CS trends in this region; rather, disincentivizing healthcare system interactions and unsatisfactory relationships with healthcare providers were thought to be the main factors for women to choose CS without indication^8,9^. We sought to explore whether digital interventions could improve the low-risk CS rate in the context of childbirth medicalization. The present study assesses the nulliparous, term, singleton, vertex (NTSV) CS delivery rate before and after the first implementation of the Smart Birth Center in a medium-sized maternity hospital in Taiwan.

## Results

### Trends in cesarean delivery rate

**Fig. 1:**
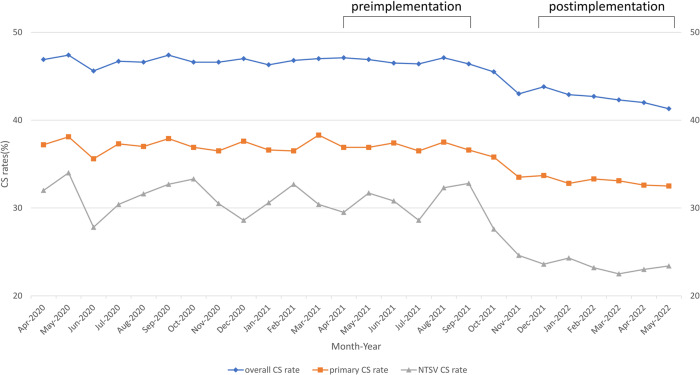
Cesarean delivery rates. The Cesarean delivery rates were calculated for each month from April 2020 to May 2022. Overall CS rate represent total number of CS deliveries divided by total number of births. Primary CS rate represent number of CS deliveries among women with no previous cesarean delivery divided by number of births among women with no previous cesarean delivery. NTSV CS rate represent number of CS deliveries in NTSV population divided by number of births in NTSV population. CS cesarean section, NTSV nulliparous term singleton vertex.

From April 2020 to May 2022, a total of 7324 live births occurred at the study hospital, 3330 (45.5%) of those were cesarean births. Trends in cesarean delivery were examined in relation to the total number of live births within the population (Fig. 1). The overall CS rate, primary CS rate, and NTSV CS rate all showed a downward trend after the implementation of the smart intrapartum surveillance system, among which NTSV CS rate demonstrated the most significant.

### Influence of interventional measure on cesarean section rate

During the study period, 3648 women admitted for delivery were included in the analysis. Of the studied deliveries, 1760 occurred prior to the implementation of the smart intrapartum surveillance system, and 1888 occurred after the implementation of the system. Table 1 lists the childbirth status of all pregnant women, women with no previous cesarean delivery, and the proportion of parturient women with NTSV status, which was determined on the basis of the Robson classification system for childbirth status. The proportion of all deliveries that were NTSV deliveries did not differ considerably between the preimplementation (21.7%) and postimplementation periods (22.9%). The absolute and relative contributions of NTSV to CS rate decreased from 6.7% (absolute contribution) and 14.3% (relative contribution) during the preimplantation period to 5.4% (absolute contribution) and 12.6% (relative contribution) during the postimplementation period. The CS rate for the NTSV population was 31.0% and 23.3% during the preimplementation and postimplementation periods, respectively, indicating a significant 24.8% (*p* = 0.014) reduction in NTSV CS rate after the implementation of the smart intrapartum surveillance system (relative risk [RR], 0.75; 95% confidence interval, 0.71–0.80). Consequently, the primary CS rate also decreased from 37.0 to 33.0% and the overall CS rate of the unit decreased from 46.8 to 42.5%, indicating a significant 10.8% (*p* = 0.022) reduction in primary CS rate (RR, 0.89; 95% CI, 0.81–0.98) and a significant 9.2% (*p* = 0.009) reduction in overall CS rate (RR, 0.91; 95% CI, 0.88–0.95) after the implementation of the smart intrapartum surveillance system (Table 2).

**Table 1:** Childbirth status of all parturient women, women without previous cesarean delivery, and NTSV population before and after implementation of smart intrapartum surveillance system.

Childbirth status	Preimplementation			Postimplementation		
	All	no CS History	NTSV	All	no CS History	NTSV
No. of births	1760	1441	381	1888	1552	433
No. of vaginal births	937	908	263	1086	1040	332
No. of CS births	823	533	118	802	512	101
Group size (%)^a^	100	81.9	21.7	100	82.2	22.9
CS rate (%)^b^	46.8	37.0	31.0	42.5	33.0	23.3
Absolute contribution to CS rate^c^	46.8	30.3	6.7	42.5	27.1	5.3
Relative contribution to CS rate^d^	100	64.8	14.3	100	63.8	12.6

*NTSV* nulliparous term singleton vertex, *CS* cesarean section.

^a^(Number of births in group population)/(total numbers of births) × 100.

^b^(Number of CS deliveries in group population)/(number of births in group population) × 100.

^c^(Number of CS deliveries in group population)/(total number of births) × 100.

^d^(Number of CS deliveries in group population)/(total number of CS deliveries) × 100.

**Table 2:** Cesarean section rates of all parturient women, women without previous cesarean delivery, and NTSV population before and after implementation of the smart intrapartum surveillance system.

Group	Preimplementation (%)	Portimplementation (%)	Variation	RR^a^ (95% confidence interval)	*p* value*
NTSV	31.0	23.3	−24.8%	0.75 (0.71–0.80)	0.0142
primary CS^b^	37.0	33.0	−10.8%	0.89 (0.81–0.98)	0.0218
All	46.8	42.5	−9.2%	0.91 (0.88–0.95)	0.0093

*NTSV* nulliparous term singleton vertex, *CS* cesarean section.

^a^RR: Relative rate (preimplementation period used as reference).

^b^(The number of women having a first cesarean delivery)/(all women giving birth who have never had a cesarean delivery).

*A *p* value of 0.05 indicates a statistically significant difference. Chi-squared test was conducted to compare groups.

### Cesarean section rate changes based on indications

**Fig. 2:**
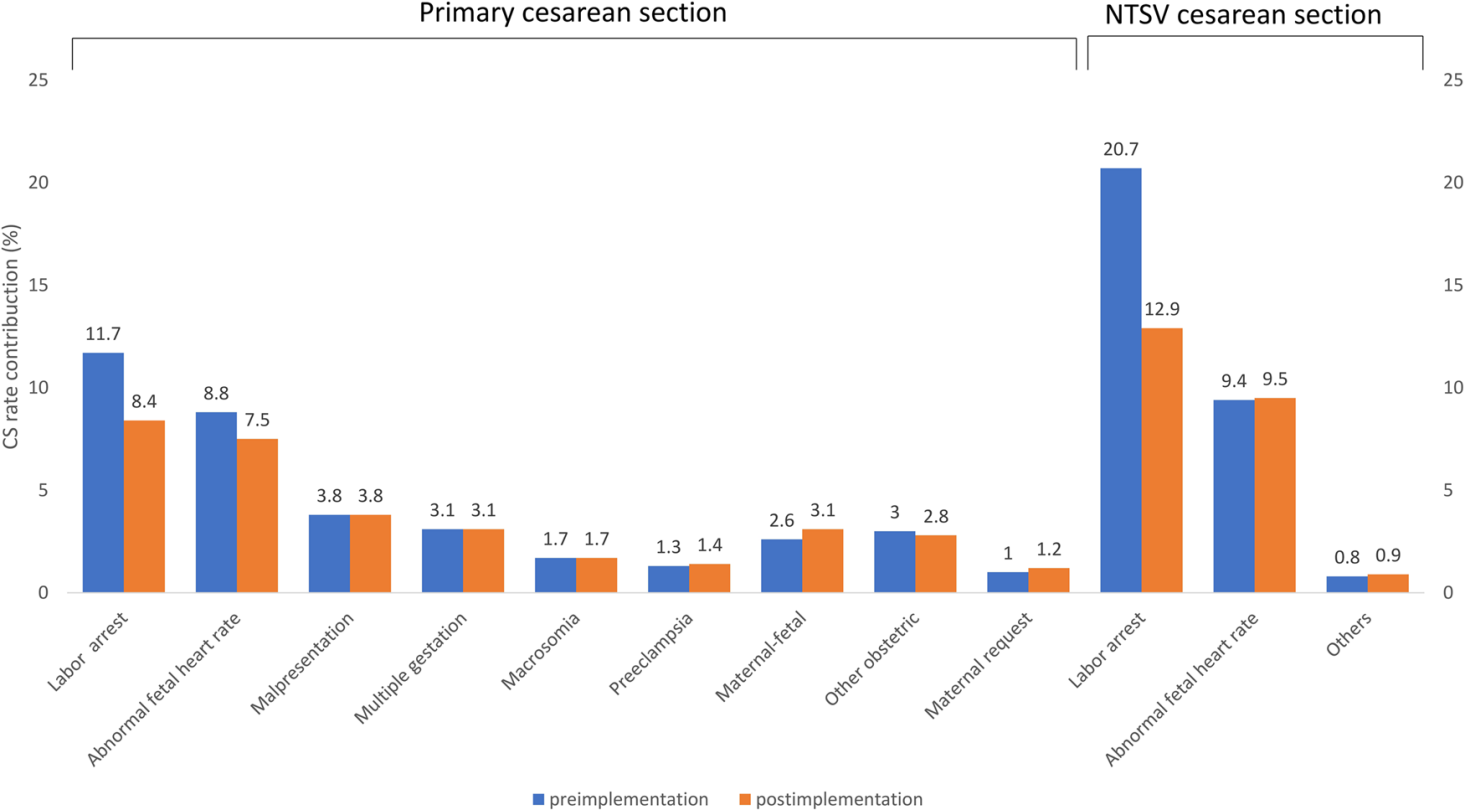
Contribution of each indication to cesarean deliveries. Contribution of each indication to primary cesarean section and NTSV cesarean section between preimplementation (blue bar) and postimplementation (orange bar) periods. CS cesarean section, NTSV nulliparous term singleton vertex.

This study examined specific indications that contributed to the decrease in CS rate. Among documented CS indications, only labor arrest decreased significantly from preimplementation period to postimplementation period for both primary CS rate contribution (11.7% vs. 8.4%; *p* = 0.003) and NTSV CS rate contribution (20.7% vs. 12.9%; *p* = 0.003) (Fig. 2). Table 3 lists the maternal demographic characteristics and obstetric outcomes for all births, vaginal births, and CS births in the NTSV population before and after the implementation of the smart intrapartum surveillance system. The vaginal birth and CS delivery groups of the NTSV population did not differ in terms of maternal age before and after the implementation of the smart intrapartum surveillance system intervention. The changes in the time duration from the admission of parturient women to delivery following the implementation of the smart intrapartum surveillance system are as follows: increase from 9.2 ± 4.2 h to 12.3 ± 5.3 h for all NTSV births, increase from 9.8 ± 4.9 h to 12.6 ± 6.1 h for NTSV vaginal births, and increase from 7.8 ± 3.8 h to 11.3 ± 5.2 h for CS NTSV births; these changes all represent a significant prolongation of the intrapartum period (all *p* < 0.0001). After the smart intrapartum surveillance system was implemented, the average gestational age of delivery was significantly prolonged from 38.5 ± 1.5 weeks to 38.8 ± 1.6 weeks for all NTSV births (*p* = 0.006) and from 38.2 ± 1.6 weeks to 39.1 ± 2.2 weeks for CS NTSV births (*p* < 0.001). Among the total birth, vaginal birth and CS birth groups in the NTSV population, no significant difference was detected in terms of length of maternal hospital stay, newborn weight, neonatal Apgar score, and the occurrence of the following: composite neonatal adverse outcome indicator, neonatal intensive care unit admission, neonatal meconium aspiration, chorioamnionitis, shoulder dystocia, perineal laceration, placental abruption, postpartum hemorrhage, maternal blood transfusion, and hysterectomy before and after the implementation of the smart surveillance system.

**Table 3:** Obstetric outcomes for the NTSV population before and after implementation of the smart intrapartum surveillance system.

Variable	NTSV population		*p* value	NTSV vaginal births		*p* value	NTSV cesarean sections		*p* value
	Preimplementation (*n* = 381)	Postimplementation (*n* = 433)		Preimplementation (*n* = 263)	Postimplementation (*n* = 332)		Preimplementation (*n* = 118)	Postimplementation (*n* = 101)	
Maternal age (mean [SD], year)	28.3 (5.1)	28.5 (4.7)	0.5607	28.5 (5.5)	28.4 (3.9)	0.7956	27.8 (4.4)	28.8 (4.8)	0.1004
Gestational age (mean [SD], week)	38.5 (1.5)	38.8 (1.6)	0.006	38.6 (2.1)	38.7 (2.1)	0.5643	38.2 (1.6)	39.1 (2.2)	0.0006
Admission to delivery time (mean [SD], h)	9.2 (4.2)	12.3 (5.3)	<0.0001	9.8 (4.9)	12.6 (6.1)	<0.0001	7.8 (3.8)	11.3 (5.2)	<0.0001
Length of maternal hospital stay (mean [SD], day)	4.3 (2.0)	4.4 (2.0)	0.4768	3.9 (2.1)	4.1 (1.8)	0.2118	5.1 (1.9)	5.3 (2.2)	0.4711
Newborn weight (mean [SD], g)	3133 (468)	3128 (501)	0.8836	3134 (379)	3126 (391)	0.8017	3131 (411)	3135 (396)	0.9225
Apgar score (5 min <7 *n* [%])	7 (1.8)	7 (1.6)	0.8092	4 (1.5)	5 (1.5)	0.9882	3 (2.5)	2 (2.0)	0.7808
Occurrence of composite NAOI (*n* [%])	12 (3.1)	13 (3.0)	0.9340	7 (2.7)	9 (2.7)	0.9706	5 (4.2)	4 (4.0)	0.9409
NICU admission (*n* [%])	52 (13.6)	55 (12.7)	0.6903	35 (13.3)	42 (12.7)	0.8126	17 (14.4)	13 (12.9)	0.7424
Meconium aspiration (*n* [%])	61 (16.0)	67 (15.5)	0.8337	39 (14.8)	51 (15.4)	0.8572	22 (18.6)	16 (15.8)	0.5830
Chorioamnionitis (*n* [%])	45 (11.8)	57 (13.2)	0.5610	32 (12.2)	44 (13.2)	0.6938	13 (11.0)	13 (12.9)	0.6731
Shoulder dystocia (*n* [%])	2 (0.5)	2 (0.5)	0.8979	2 (0.8)	2 (0.6)	0.8149			
Third and fourth degree laceration (*n* [%])	2 (0.5)	2 (0.5)	0.8979	2 (0.8)	2 (0.6)	0.8149			
Placenta abruption (*n* [%])	2 (0.5)	1 (0.2)	0.4900				2 (1.7)	1 (1.0)	0.6554
Postpartum hemorrhage (*n* [%])	34 (8.9)	40 (9.2)	0.8765	23 (8.7)	31 (9.3)	0.8030	11 (9.3)	9 (8.9)	0.9163
Maternal blood transfusion (*n* [%])	1 (0.3)	0	0.2864	0	0		1 (0.8)	0	0.3549
Hysterectomy (*n* [%])	1 (0.3)	0	0.2864	0	0		1 (0.8)	0	0.3549

A *p* value of 0.05 indicates a statistically significant difference. For significance testing, chi-squared test and *t* test were used for categorical and continuous variables, respectively.

*NTSV* nulliparous term singleton vertex, *SD* standard deviation, *NAOI* neonatal adverse outcome indicator, *NICU* neonatal intensive care unit.

## Discussion

The results of this study indicate that the introduction of the smart intrapartum surveillance system resulted in a significant decrease in the primary CS rate for low-risk NTSV pregnancies and an improvement in the overall CS rate. Perinatal outcomes did not differ significantly between preintervention period and postintervention period; however, for pregnant women, the average time from admission to delivery increased after the implementation of the intrapartum surveillance system.

CS is a major surgery that saves the lives of mothers and fetuses when physiological vaginal delivery is no longer the safest option, and it is now the most common surgical procedure performed on women^5,10^. However, from a public health perspective, performing CSs for low-risk pregnancies increases short-term and long-term health risks for mothers and fetuses^10^. Therefore, the CS rate of NTSV pregnancies is defined as an important quality indicator of obstetric care^11,12^. NTSV CS rates vary widely throughout the world and among medical institutions, and physicians and nursing care providers have indicated that clinical practice patterns in labor and delivery units affect the number of CSs performed^13,14^. Various intrapartum care methods for reducing the rate of NTSV CS births have been proposed, and they mainly focus on labor management and fetal heart rate interpretation for care providers^10,11,15,16^. Consistent with the methods adopted by other studies, the present study adopted interventions and focused on labor management for care providers and the awareness of pregnant women regarding physiologic birth parameters that encourage vaginal birth, and they were effective in reducing primary CS birth rates.

In the present study, we implemented a smart intrapartum surveillance system that simulates and displays labor course progress through the digital analysis of intrapartum maternal birth parameters data, serves as an early warning system for physician staff and nursing caregivers, and provides medical counseling for pregnant women. Several population-based studies have reported that, for NTSV pregnancies, the most common indication for CS delivery is labor dystocia or an arrest of labor, both of which suggest that more objective and standardized approaches should be used^17^. Notably, the definition of an arrest of labor should be revised because a growing body of evidence indicates that labor progresses at a substantially slower rate relative to that defined in various studies^18,19^. The algorithms employed by the smart intrapartum surveillance system are based on the WHO Labor Care Guide, which redefines the duration of the first and second stages of labor and provides guidance on the timing and use of labor interventions for improving the health and well-being of women and their babies^20^. The results of the present study are consistent with those of other studies, indicating that increasing the adherence of health-care workers to the criteria for arrest of labor significantly reduced the rate of NTSV CS delivery from 31.0% to 23.3% after an electronic intervention was implemented in the labor and delivery unit of a maternity hospital in Taiwan.

The implementation of an electronic intrapartum surveillance system in the present study effectively reduced NTSV CS rates, and this was achieved through the digital analysis of birth parameters and the reduction of the diagnosis of labor dystocia for patients with NTSV status. Electronic surveillance systems have been widely deployed in various medical care settings (especially emergency and critical care units) to assist physicians in providing efficient patient care^21,22^. An automated health surveillance system assists medical care personnel in making clinical decisions on the basis of three fundamental frameworks, namely the collection of comprehensive data through wearable devices and electronic medical records, the rapid analysis of real-time data, and the timely issuance of early warnings regarding patient health risks. Studies have reported the successful implementation electronic surveillance systems in clinical settings to predict severe sepsis in emergency and intensive care units and to improve glucose management in operating rooms^21–24^.

A hospital labor and delivery unit is responsible for providing comprehensive maternal fetal health care from the intrapartum period and delivery to the postpartum period. In a clinical setting, health-care workers must manage the rapid progress of maternal fetal vital signs and dynamic labor courses at all times, and they must monitor these situations to ensure that the appropriate management responses are made. In the present study, an electronic maternal surveillance system based on the MEWC was developed, and it was implemented in a labor and delivery unit to improve the clinical recognition of maternal deterioration. In addition to the digital monitoring of maternal fetal vital signs and the timely issuance of early warnings and prompt alerts^25^, our smart intrapartum surveillance system can also serve as a labor management program. With the application of the labor progress algorithms that are integrated into the smart intrapartum surveillance system, which was developed on the basis of American College of Obstetricians and Gynecologists and WHO criteria, labor dystocia alerts can be sent to the nursing station dashboard; this system frees up time for health-care workers in labor and delivery units to implement birth education, comfort measures, and labor support for mothers. Additionally, the electronic partograph algorithm of the system converts birth parameter data into graphical representations of labor progress that visualize maternal cervical dilation and fetal descent over time and are presented on patient bedside information terminals. In a clinical setting, the partogram can help obstetrical care providers to engage in bidirectional communication, share decision-making responsibilities, and provide medical consultation and psychological support to intrapartum mothers. Literature findings support the implementation of these interventions, which are based on electronic partographs and are key factors that enable safe primary CS delivery for NTSV pregnancies^26^.

More than a century ago, William Osler described medicine as “a science of uncertainty and an art of probability.” The complexity and unpredictability of medicine influence the work of obstetricians and nursing staff in labor and delivery units, where healthy patients with high expectations for their future are being cared for and where the incidences of malpractice lawsuits, defensive medical care, and unnecessary CS deliveries are high. Computational medicine has developed, with the objective for advancing health care by developing computational models for diseases, personalizing these models using patient data, and applying these models to improve the diagnosis and treatment of diseases; for obstetricians, these models assist in decision-making pertaining to maternity care^27^. The expansion of computational maternal fetal medicine comes with benefits, but also potential adverse effects, including those to economic, psychological, societal well-being and further exacerbation of gender inequalities^28,29^. The development of this type of digital health intervention should also tack digital health’s gender inequities through feminist intersectionality framework so that they can be prevented and mitigated^30^. In addition, implementing such a smart medical system should go beyond too little, too late, too much, too soon scenario and pursue a pathway towards evidence-based, respectful maternity care.

We developed a successful model that applies a smart intrapartum surveillance system and an electronic partograph algorithm to reduce the number of primary CS births. Through multidisciplinary research conducted to solve the key problems that affect health-care stakeholders (e.g., academia, hospital systems, and policy makers), the substantial benefits of obstetrical computational medicine can be realized and promoted, thereby transforming maternal and fetal health care. The algorithms used in the present study were developed on the basis of up-to-date guidelines, and the pre–post comparative research results indicates that they can be a decisive obstetric intervention. In addition, the data collected in the present study were automatically extracted from the electronic medical records of an institution to avoid the bias resulting from manual data collection.

The present study has several limitations. The main findings were obtained on the basis of data from a single institution; therefore, their generalizability is limited. Because hospitals have different equipment structures, cultural backgrounds, training systems, and manpower allocation frameworks, they often differ considerably in terms of their CS rates. The postinterventional reductions in the overall CS rate (−9.2%), primary CS rate (−10.8%), and NTSV CS rate (−24.8%) of the unit examined in the present study are not necessarily replicable in other hospitals. Additionally, the retrospective design of the present study limited our ability to clarify the mechanism of action of each algorithm in the context of maternal–fetal care and labor management; therefore, more prospective studies are required to further clarify user perceptions and the effect of each algorithm on the behavior of health-care providers. Another limitation pertains to the finding that the average length of intrapartum stay in the labor and delivery unit was increased; specifically, no statistical analysis was performed to clarify the association between the average length of intrapartum stay and medical cost. In addition to considering the short-term and long-term maternal–fetal health effects of CS delivery, future studies should also incorporate the aforementioned influencing factors and explore differences in overall medical expenditure to assess the overall effects and benefits of implementing a smart intrapartum surveillance system in a hospital labor and delivery unit.

In conclusion, a hospital labor and delivery unit that provides maternal–fetal health care and labor management is typically a diverse, variable, and unpredictable environment where a diverse range of comorbidities are being managed. We propose a smart intrapartum surveillance system as a novel monitoring model for labor and delivery units. The system is designed to aggregate and summarize individual maternal, fetal, and labor progress information and visually display it on the nursing station dashboard and patient bedside terminals to provide an early warning and clinical decision-making tool for the staff of a labor and delivery unit. The present study revealed that the use of the smart intrapartum surveillance system in a labor and delivery unit effectively reduced the NTSV CS delivery rates of the unit.

## Methods

### Study design

This retrospective cross-sectional study examined the labor and delivery care provided to parturient women at a local medium-sized maternity hospital in Taoyuan City, Taiwan (Hungchi Women & Children’s Hospital). On average, this maternity hospital manages approximately 3000 births annually. It has 50 beds (including 10 predelivery beds and two puerperium beds), two delivery rooms, and one CS room that is located in its labor and delivery unit. The labor and delivery staff comprise 12 physicians and 32 midwives and nursing staff who work shifts. Each pregnant woman has her designated attending physician responsible for supervising care and on call for delivery. One of the attending physician on duty stays in the labor and delivery unit to handle emergency notes at any time. Each pregnant woman has a responsible nurse in charge of the first-line care in three shifts, and the ratio of nurses to pregnant women during the day shift is 1:1.

The present study was conducted between April 2021 and May 2022. The research data pertaining to the period before the implementation of the smart intrapartum surveillance system were collected between April 2021 and September 2021, and those pertaining to the postimplementation period were collected between December 2021 and May 2022. The period from mid-October 2021 to November 2021 was regarded as a transitionary period for system implementation; thus, it was excluded from the analysis. Retrospective data on all births, including mode of delivery, were collected from April 2020 to serve as a basis for comparison of study period. Research data were collected from the electronic medical records of labor and delivery unit.

### Implementation phases

The three phases of this study were constructed by reviewing and sorting out relevant previous clinical literatures to assist in carrying out design thinking innovation, and completing prototype system development as well as the final usability testing and evaluation. In the first phase, we systematically searched for the scientific articles to identify the main trends of the development of digital user interfaces and AI algorithms in the smart health care system. We examined the possibilities for application in the current usage context through the compilation of relevant literatures. Later, the procedures were followed by the evaluation of the clinical intrapartum care workflow and maternal fetal health data transfer process within the study institution and inviting institution’s health care providers to conduct the interviews. In addition to listening their experience and needs in maternal fetal health care and labor management, we exchanged ideas on the use of innovative technologies in the intrapartum healthcare system as input for the design of the smart intrapartum surveillance system prototype system, which was then completed. In the second phase, we invited hospital maternity health care providers, nursing staffs, information engineers who assisted in the program prototype design in the previous phase. Five out-of-hospital maternal fetal medicine experts, and two human factors professionals to conduct a usability test on the system starting from the context of use requirements and user experiences. We sorted out the improvement opinions of real users and professional experts on the system workflows and interface design to improve the system prototype, thus determining the final smart intrapartum surveillance system and Smart Birth Center architecture for implementation. In the third phase, after the transition period of this study, the smart intrapartum surveillance system was formally used in labor and delivery unit of the hospital to assist intrapartum maternal fetal health care and labor management, while study participants and research data collection was conducted following the guidelines of the Helsinki Declaration.

### Smart Birth Center architecture

**Fig. 3:**
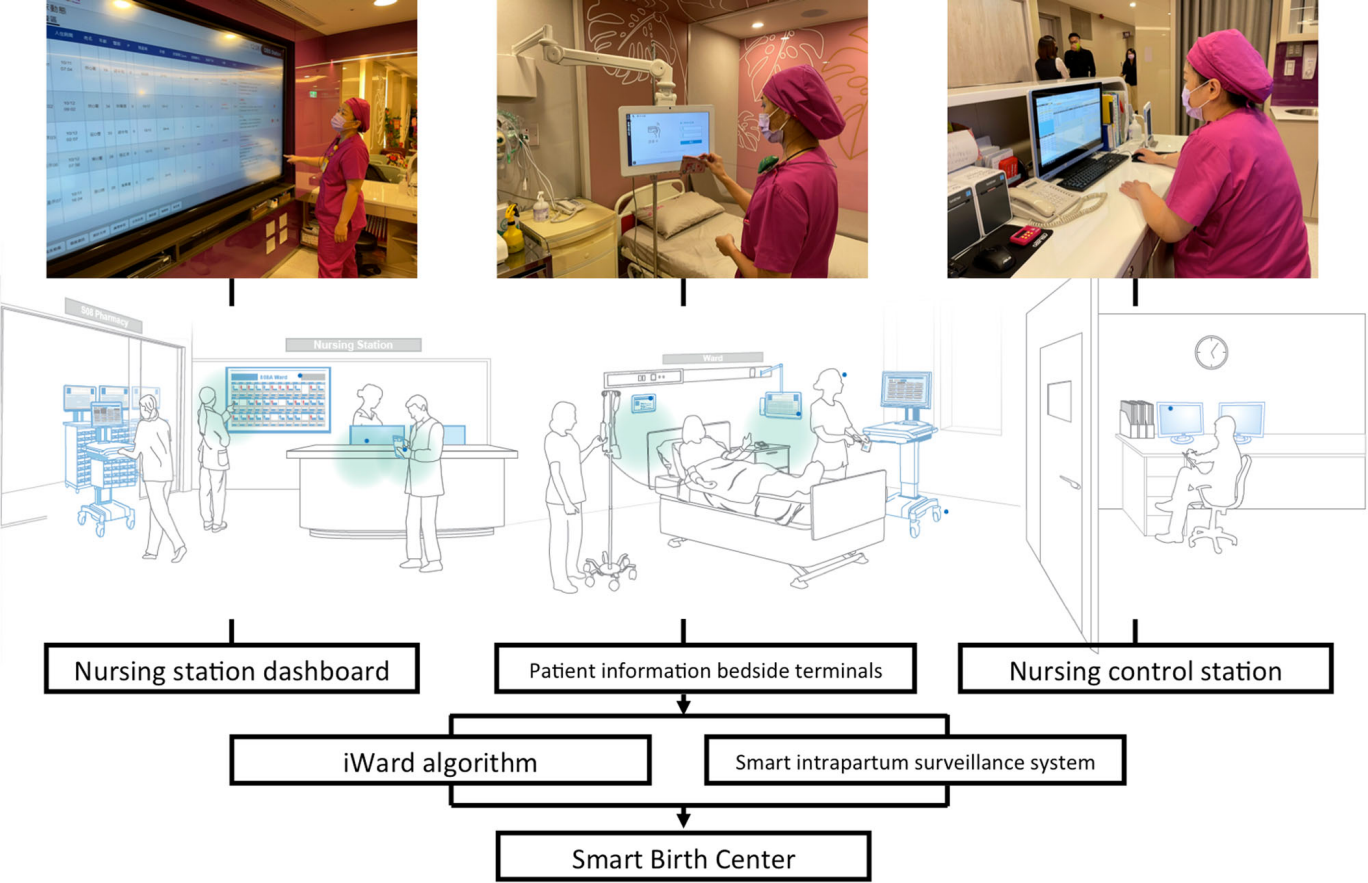
Smart Birth Center architecture. The Smart Birth Center is a secure intranet-based multifunction hardware and software architecture that connects a nursing control station, a nursing station dashboard, and multiple patient information bedside terminals through an iWard algorithm and smart intrapartum surveillance system. Written informed consents have obtained from all persons appearing in the photographs for the publication of them.

The Smart Birth Center is a secure, intranet-based, and multifunction hardware and software architecture that connects a nursing control station, a nursing station dashboard, and multiple patient information bedside terminals through an iWard algorithm and the smart intrapartum surveillance system (Fig. 3; the person in the photographs related to Smart Birth Center consented to appear in this article and associated publications of them). The Smart Birth Center first receives, integrates, summarizes, and analyzes the data obtained from physiologic monitors, electronic health records (EHRs), laboratory systems, and the nursing control station (data from the station are human input data); subsequently, it transmits and displays the relevant information on the nursing control station, nursing station dashboard, patient bedside information terminals, and the personal portable display devices of health-care providers.

### Smart intrapartum surveillance system

**Fig. 4:**
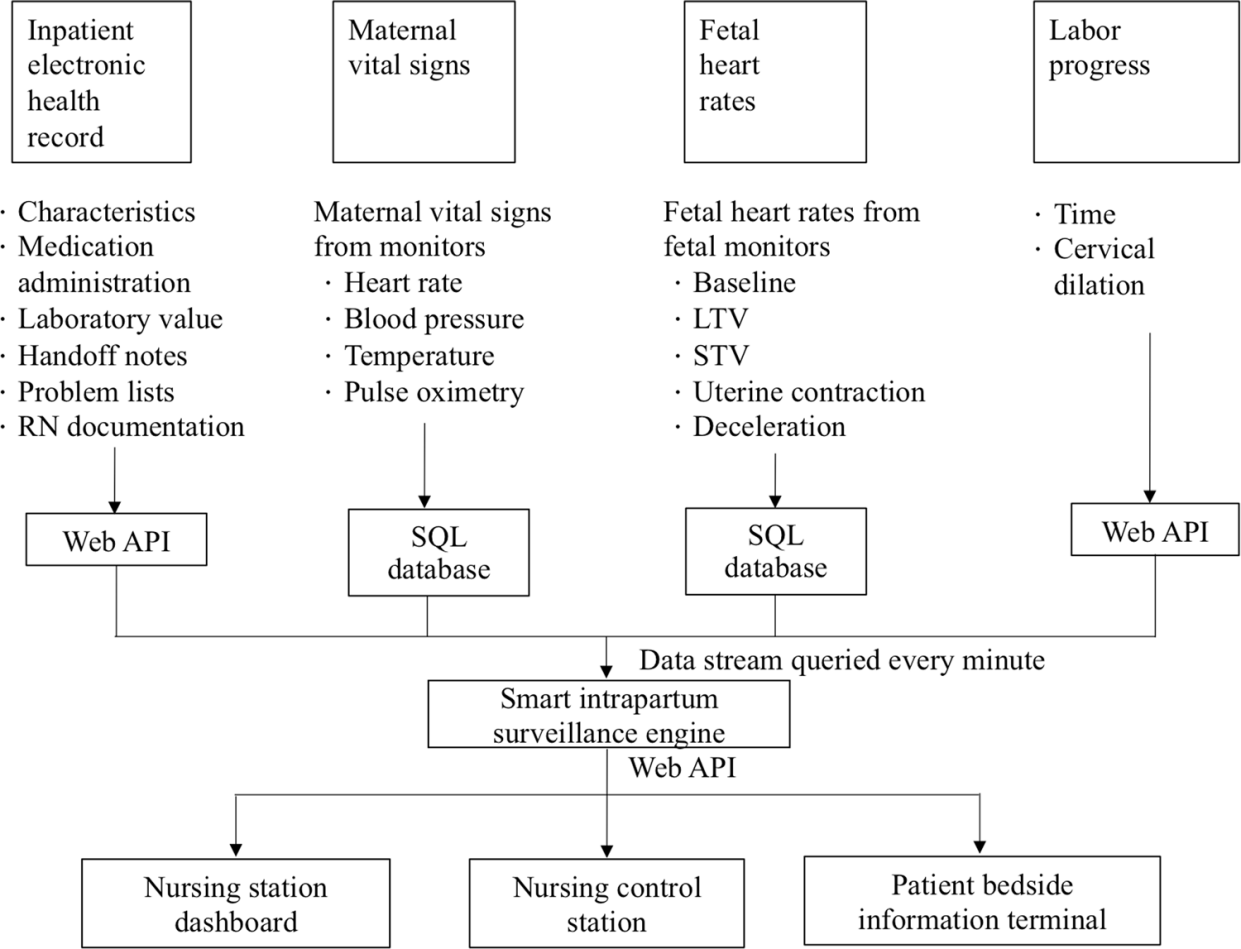
Data flow diagram. Information from electronic health records, bedside maternal and fetal vital sign monitors, and labor progress data are queried every minute by a surveillance engine. The aggregated data are then displayed on a nursing control station, a nursing station dashboard, and patient information bedside terminals through a web interface. API application programming interface, SQL structured query language, RN registered nurse, LTV long-term variation, STV short-term variation.

The smart intrapartum surveillance system is a computer knowledge-based clinical decision support system (CDSS) that analyzes data obtained from EHRs (i.e., maternal vital signs, fetal heart rate, and labor progress course) and provides prompts and reminders to help health-care providers to apply evidence-based clinical guidelines at the point of intrapartum care. The system reflects the data stream of multiple patients in near-real time. The types of patient data processed by the system include electronic medical records, maternal vital signs, fetal heart rate, labor progress, and physician scheduling, and they are all maintained by the Smart Birth Center (Fig. 4). Three software algorithms were developed and embedded into the smart intrapartum surveillance system to serve as a CDSS for maternal health care, fetal health care, and individualized labor and delivery care. Through a customizable paging alert system, these algorithms can automatically send a reminder to the staff of the labor and delivery unit to reevaluate patients whose conditions warrant additional intervention. The maternal vital signs, fetal vital signs, and labor progress alerts of the system were set in accordance with the AIM-Maternal early warning signs protocol (2015)^31^, FIGO-Consensus guidelines on intrapartum fetal monitoring cardiotocography (2015)^32^, and WHO Labor Care Guide (2020)^20^, respectively (Table 4). When the clinical data exceeded the reference thresholds, a small bell alert icon is displayed on the nursing control station, nursing dashboard, and patient information terminals to alert station or bedside nurses. A staff member can tap the icon to verify the abnormal data, which are presented in another small popup window that expands on the screen. An additional method for implementing smart intrapartum decision support is the provision of an electronic partograph; this allows for continuous labor progress monitoring, which is achieved by examining cervical dilation over time through the screen of a patient bedside information terminal. The algorithm of the electronic partograph applies evidence-based time limits for each centimeter of cervical dilation during the active first and second stages of labor; the starting point of the active first stage of labor is a cervical dilation of 5 cm (Table 4). The electronic partograph is designed to enable health-care providers and parturient women to engage in bidirectional communication regarding clinical intervention, consultation, and decision-making, thereby improving the overall childbirth experience.

**Table 4:** Reference threshold for the smart intrapartum surveillance system.

*Maternal vital sign*	
Pulse (bpm)	Alert: <60, ≥120
Systolic blood pressure (mmHg)	Alert: <80, ≥140
Diastolic blood pressure (mmHg)	Alert: ≥90
Temperature (°C)	Alert: <35.0, ≥37.5
Oximetry (%)	Alert: <95
*Fetal heart rate*	
Baseline (bpm)	Alert: <110, ≥160
Deceleration	Alert: Late deceleration
*Labor progress*	
First stage	
Cervical dilation	Alert: 5 cm ≥ 6 h, 6 cm ≧ 5 h 7 cm ≥ 3 h, 8 cm ≥ 2.5 h, 9 cm ≥ 2 h
*Second stage*	
Nulliparous women	Alert: ≥3 h
Multiparous women	Alert: ≥2 h

*bpm* beats per minute.

### Data collection

The present study collected data on births involving women with NTSV status (i.e., nulliparous status [first time giving birth], term gestation status [gestational age of 37.0 weeks or longer], singleton status [one fetus], and vertex status [head-down position]) in an institutional labor and delivery unit before (preimplementation) and after (postimplementation) the implementation of the smart intrapartum surveillance system. The primary outcomes were overall CS rate, primary CS rate, and the CS rate for the NTSV population. Overall CS rate is calculated as the number of cesarean births divided by total live births. The primary CS rate is the number of women having a first cesarean delivery divided by all women giving birth who have never had a cesarean delivery (the sum of primary cesarean and vaginal births without a previous cesarean). The NTSV CS rate is the number of CS deliveries in NTSV group divided by number of births in NTSV population. Indication for these cesarean deliveries were also documented in this research, that included labor arrest (included failed operative vaginal deliveries), abnormal or indeterminate fetal heart rate, malpresentation, multiple gestation, suspected macrosomia, preeclampsia, maternal-fetal indication (maternal indications include all maternal conditions that predated the pregnancy and fetal indications included congenital anomalies and growth restriction), other obstetric conditions (specific to the current pregnancy, such as placenta previa, abruption, or cord prolapse), and maternal request. The secondary outcomes were maternal age, gestational age at time of delivery, admission to delivery time, length of maternal hospital stay, birth weight, 5-min Apgar score, occurrence of composite neonatal adverse outcome indicator (with one or more of 15 diagnosis and 7 procedures)^33^, the incidence of neonatal intensive care unit (NICU) admission within the first 24 h after birth, meconium aspiration syndrome (the neonatal respiratory distress that occurs in the context of meconium-stained amniotic fluid), chorioamnionitis (an infection with resultant inflammation of any combination of the amniotic fluid, placenta, fetus, fetal membranes, or decidua), shoulder dystocia, third-and-fourth degree laceration, placenta abruption, poatpartum hemorrhage (greater than 500 mL estimated blood loss associated with vaginal delivery or greater than 1000 mL estimated blood loss associated with cesarean delivery), and maternal transfusion. Data were also collected from EHRs. Permission was granted by the implementation site for the present study, and the proper security measures and passwords were provided by the relevant organization. The collected data were deidentified to protect the confidentiality of patients and ensure compliance with the Health Insurance Portability and Accountability Act policies. The present study was a quality improvement project and no risk was posed to human participants (the collected data did not contain any patient identifiers). The Research Ethics Committee of the Taiwan Maternal Fetal Medicine Society (TMFMS-REC-20-1018A) approved this study.

### Statistical analysis

Medcalc software version 20.009 was employed for all analyses (Medcalc Software). The means and standard deviations for continuous variables and the percentages or proportions for categorical data were calculated, *t* tests were conducted to test for the significance of continuous variables, and chi-square tests were conducted to compare the CS rates of the NTSV population before and after the implementation of the Smart Birth Center. A *p* value of <0.05 was regarded as statistically significant.

### Reporting summary

Further information on research design is available in the Nature Research Reporting Summary linked to this article.

## Supplementary information

Reporting Summary

## Data Availability

The dataset generated and analyzed during the current study are available from the corresponding author on reasonable request.
