# Supplementary material for: Reducing primary cesarean delivery rate through implementation of a smart intrapartum surveillance system
Source: NPJ Digit Med. 2023 Jul 11;6:126. doi: 10.1038/s41746-023-00867-y (PMC10336068; doi:10.1038/s41746-023-00867-y)
Supplement: Supplementary file 1 — Reporting Summary [file 41746_2023_867_MOESM1_ESM.pdf]

nature portfolio

Corresponding author(s): Po Jen Cheng
Last updated by author(s): Nov 22, 2022

Reporting Summary

Nature Portfolio wishes to improve the reproducibility of the work that we publish. This form provides structure for consistency and transparency in reporting. For further information on Nature Portfolio policies, see our Editorial Policies and the Editorial Policy Checklist.

Statistics

For all statistical analyses, confirm that the following items are present in the figure legend, table legend, main text, or Methods section.

n/a	Confirmed	
☐	☒	The exact sample size (*n*) for each experimental group/condition, given as a discrete number and unit of measurement
☐	☒	A statement on whether measurements were taken from distinct samples or whether the same sample was measured repeatedly
☐	☒	The statistical test(s) used AND whether they are one- or two-sided<br>*Only common tests should be described solely by name; describe more complex techniques in the Methods section.*
☐	☒	A description of all covariates tested
☐	☒	A description of any assumptions or corrections, such as tests of normality and adjustment for multiple comparisons
☐	☒	A full description of the statistical parameters including central tendency (e.g. means) or other basic estimates (e.g. regression coefficient) AND variation (e.g. standard deviation) or associated estimates of uncertainty (e.g. confidence intervals)
☐	☒	For null hypothesis testing, the test statistic (e.g. *F*, *t*, *r*) with confidence intervals, effect sizes, degrees of freedom and *P* value noted<br>*Give P values as exact values whenever suitable.*
☒	☐	For Bayesian analysis, information on the choice of priors and Markov chain Monte Carlo settings
☒	☐	For hierarchical and complex designs, identification of the appropriate level for tests and full reporting of outcomes
☒	☐	Estimates of effect sizes (e.g. Cohen's *d*, Pearson's *r*), indicating how they were calculated

Our web collection on statistics for biologists contains articles on many of the points above.

Software and code

Policy information about availability of computer code

Data collection	N/A
Data analysis	All data were analyzed using Medcalc software version 20.009.

For manuscripts utilizing custom algorithms or software that are central to the research but not yet described in published literature, software must be made available to editors and reviewers. We strongly encourage code deposition in a community repository (e.g. GitHub). See the Nature Portfolio guidelines for submitting code & software for further information.

Data

Policy information about availability of data

All manuscripts must include a data availability statement. This statement should provide the following information, where applicable:

- Accession codes, unique identifiers, or web links for publicly available datasets
- A description of any restrictions on data availability
- For clinical datasets or third party data, please ensure that the statement adheres to our policy

The datasets generated and/or analysed during the current study are available from the corresponding author on reasonable request.

Human research participants

Policy information about studies involving human research participants and Sex and Gender in Research.

Reporting on sex and gender	This study included only pregnant participants in labor and in the term"sex" was used as a consequence.
Population characteristics	Presented in tabular form in the manuscriopt.
Recruitment	Research data were collected from the electronic medical records of labor and delivery unit.
Ethics oversight	The collected data were deidentified to protect the confidentiality of patients and ensure compliance with the Health Insurance Portability and Accountability Act policies. The present study should be exempted from a formal institutional review board review because it was a quality improvement project and no risk was posed to human participants (the collected data did not contain any patient identifiers).

Note that full information on the approval of the study protocol must also be provided in the manuscript.

Field-specific reporting

Please select the one below that is the best fit for your research. If you are not sure, read the appropriate sections before making your selection.

☒ Life sciences ☐ Behavioural & social sciences ☐ Ecological, evolutionary & environmental sciences

For a reference copy of the document with all sections, see nature.com/documents/nr-reporting-summary-flat.pdf

Life sciences study design

All studies must disclose on these points even when the disclosure is negative.

Sample size	During the study period, 3648 women admitted for delivery were included in the analysis. Of the studied deliveries, 1760 occurred prior to the implementation of the smart intrapartum surveillance system, and 1888 occurred after the implementation of the system.
Data exclusions	The present study was conducted between April 2021 and May 2022. The research data pertaining to the period before the implementation of the smart intrapartum surveillance system were collected between April 2021 and September 2021, and those pertaining to the postimplementation period were collected between December 2021 and May 2022. The period from mid-October 2021 to November 2021 was regarded as a transitionary period for system implementation; thus, it was excluded from the analysis.
Replication	N/A
Randomization	N/A
Blinding	N/A

Reporting for specific materials, systems and methods

We require information from authors about some types of materials, experimental systems and methods used in many studies. Here, indicate whether each material, system or method listed is relevant to your study. If you are not sure if a list item applies to your research, read the appropriate section before selecting a response.

Materials & experimental systems

n/a	Involved in the study	
☒	☐	Antibodies
☒	☐	Eukaryotic cell lines
☒	☐	Palaeontology and archaeology
☒	☐	Animals and other organisms
☐	☒	Clinical data
☒	☐	Dual use research of concern

Methods

n/a	Involved in the study	
☒	☐	ChIP-seq
☒	☐	Flow cytometry
☒	☐	MRI-based neuroimaging

Clinical data

Policy information about clinical studies
All manuscripts should comply with the ICMJE guidelines for publication of clinical research and a completed CONSORT checklist must be included with all submissions.

Clinical trial registration	N/A
Study protocol	The present study assesses the nulliparous, term, singleton, vertex (NTSV) CS delivery rate before and after the first implementation of the Smart Birth Center in a medium-sized maternity hospital in Taiwan.
Data collection	The present study collected data on births involving women with NTSV status (i.e., nulliparous status [first time giving birth], term gestation status [gestational age of 37.0 weeks or longer], singleton status [one fetus], and vertex status [head-down position]) in an institutional labor and delivery unit before (preimplementation) and after (postimplementation) the implementation of the smart intrapartum surveillance system. The primary outcome was the CS rate for the NTSV population. The secondary outcomes were maternal age, gestational age at time of delivery, birth weight, 5-min Apgar score, the incidence of neonatal intensive care unit (NICU) admission within the first 24 h after birth, meconium aspiration syndrome, shoulder dystocia, third-and-fourth degree laceration, and maternal transfusion. Data were also collected from EHRs. Permission was granted by the implementation site for the present study, and the proper security measures and passwords were provided by the relevant organization.
Outcomes	The results of this study indicate that the introduction of the smart intrapartum surveillance system resulted in a significant decrease in the primary CS rate for low-risk NTSV pregnancies and an improvement in the overall CS rate. Perinatal outcomes did not differ significantly between preintervention period and postintervention period; however, for pregnant women, the average time from admission to delivery increased after the implementation of the intrapartum surveillance system.
